# 1-^13^C-propionate breath testing as a surrogate endpoint to assess efficacy of liver-directed therapies in methylmalonic acidemia (MMA)

**DOI:** 10.1038/s41436-021-01143-8

**Published:** 2021-04-05

**Authors:** Irini Manoli, Alexandra R. Pass, Elizabeth A. Harrington, Jennifer L. Sloan, Jack Gagné, Samantha McCoy, Sarah L. Bell, Jacob D. Hattenbach, Brooks P. Leitner, Courtney J. Duckworth, Laura A. Fletcher, Thomas M. Cassimatis, Carolina I. Galarreta, Audrey Thurm, Joseph Snow, Carol Van Ryzin, Susan Ferry, Nicholas Ah Mew, Oleg A. Shchelochkov, Kong Y. Chen, Charles P. Venditti

**Affiliations:** 1 Medical Genomics and Metabolic Genetics Branch, National Human Genome Research Institute, National Institutes of Health, Bethesda, MD USA; 2 Diabetes, Endocrinology, and Obesity Branch, National Institute of Diabetes and Digestive and Kidney Diseases, National Institutes of Health, Bethesda, MD USA; 3 Pediatrics and Developmental Neuroscience Branch, National Institute of Mental Health, National Institutes of Health, Bethesda, MD USA; 4 Office of the Clinical Director, National Institute of Mental Health, National Institutes of Health, Bethesda, MD USA; 5 Children’s National Health System, Washington, DC USA

## Abstract

**Purpose:**

To develop a safe and noninvasive in vivo assay of hepatic propionate oxidative capacity.

**Methods:**

A modified 1-^13^C-propionate breath test was administered to 57 methylmalonic acidemia (MMA) subjects, including 19 transplant recipients, and 16 healthy volunteers. Isotopomer enrichment (^13^CO_2_/^12^CO_2_) was measured in exhaled breath after an enteral bolus of sodium-1-^13^C-propionate, and normalized for CO_2_ production. 1-^13^C-propionate oxidation was then correlated with clinical, laboratory, and imaging parameters collected via a dedicated natural history protocol.

**Results:**

Lower propionate oxidation was observed in patients with the severe *mut*^0^ and *cblB* subtypes of MMA, but was near normal in those with the *cblA* and *mut*^−^ forms of the disorder. Liver transplant recipients demonstrated complete restoration of 1-^13^C-propionate oxidation to control levels. 1-^13^C-propionate oxidation correlated with cognitive test result, growth indices, bone mineral density, renal function, and serum biomarkers. Test repeatability was robust in controls and in MMA subjects (mean coefficient of variation 6.9% and 12.8%, respectively), despite widely variable serum methylmalonic acid concentrations in the patients.

**Conclusion:**

Propionate oxidative capacity, as measured with 1-^13^C-propionate breath testing, predicts disease severity and clinical outcomes, and could be used to assess the therapeutic effects of liver-targeted genomic therapies for MMA and related disorders of propionate metabolism.

**TRIAL REGISTRATION:**

This clinical study is registered in www.clinicaltrials.gov with the ID: NCT00078078. Study URL: http://clinicaltrials.gov/ct2/show/NCT00078078

## Graphical abstract

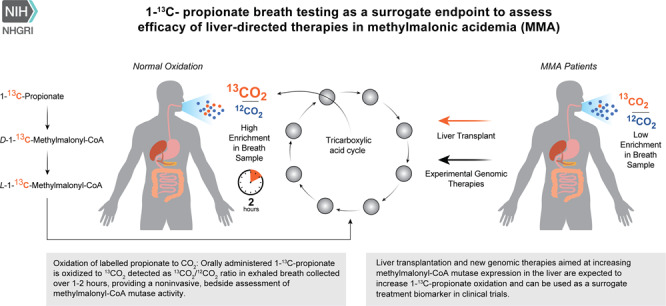

## INTRODUCTION

Isolated methylmalonic acidemia (MMA) is caused by biallelic pathogenic variants in the gene coding for the mitochondrial enzyme methylmalonyl-CoA mutase (OMIM 251000, *MMUT*) or defects in the transport and metabolism of its cofactor, 5’-deoxyadenosylcobalamin, which result in the cobalamin A (*cblA*) (OMIM 251100, *MMAA*), *cblB* (OMIM 251110, *MMAB*), and *cblD*-MMA (OMIM 277410, *MMADHC*) subtypes.^1^ The MMUT enzyme is essential for the terminal oxidation of amino acids (valine, isoleucine, methionine, threonine), odd-chain fatty acids, and cholesterol, and produces succinyl-CoA, an important Krebs cycle intermediate. MMA is characterized by recurrent episodes of metabolic ketoacidosis and multiorgan complications, variably including failure to thrive, renal disease, pancreatitis, optic nerve atrophy, movement disorders, and intellectual impairment.^2,3^ Patients who harbor variants in *MMUT* resulting in very low or absent MMUT enzyme activity are the most adversely affected (*mut*^0^ subtype), while those with partial deficiency (*mut*^−^) are associated with markedly milder phenotypes and improved survival.^4,5^ The high mortality of individuals with *mut*^0^ MMA has led to the implementation of elective liver or combined liver–kidney transplantation (LT or LKT) as a surgical therapy that can provide metabolic stability in severe patients.^6,7^ Although this approach prevents metabolic decompensations, it carries the risks of surgical complications and lifelong immunosuppression and does not completely prevent extrahepatic disease manifestations especially from the central nervous system.^7–10^

The lack of curative therapies for MMA has encouraged the development of alternative approaches to restore enzyme activity, including adeno-associated virus (AAV)-mediated gene therapy, systemic messenger RNA (mRNA) therapy, and genome editing.^11–13^ While murine models have repeatedly demonstrated that plasma metabolites, such as methylmalonic acid, can be robust markers of hepatic correction, in MMA patients, serum and urine methylmalonic acid, propionylcarnitine, and 2-methylcitric acid can be highly variable, between and within patients, and directly influenced by dietary protein intake, the gut microbiome, and renal function.^14–17^ Furthermore, and in contrast to other disorders such as maple syrup urine disease, metabolites remain massively elevated after successful liver transplantation. Given that mouse models of MMA demonstrated increased 1-^13^C-propionate oxidation in response to liver-directed *MMUT* gene therapy, even in the setting of an expanded circulating methylmalonic acid pool, we sought to explore the use of stable isotopes to quantify in vivo hepatic MMUT function in MMA patients.

Stable isotope tracers provide a safe, nonradioactive analysis of the metabolic fate of a specific compound at the whole-body level, and can be administered to patients of all age groups, including newborns.^18–20^ Early studies performed in a small number of patients with methylmalonic and propionic acidemia (MMA, PA) used intravenous administration of radiolabeled 1-^14^C-valine or propionate to demonstrate that the metabolism of labeled propionate to ^14^CO_2_ was rapid in controls, but severely diminished in patients with propionate metabolic disorders.^21,22^ Subsequently, the nonradioactive isotopomer, 1-^13^C-propionate, was used to confirm the observations noted with radiolabels.^17,23–27^ These studies concluded that in vivo propionate oxidation was a better predictor of clinical severity than in vitro enzyme activity measurements, plasma methylmalonic acid levels, and/or urinary MMA excretion.^25,26^ A single oral bolus method using 1-^13^C-propionate showed equivalent performance to intravenous administration, but did not correlate with disease severity in four *mut* MMA and four *cblA* subjects.^17,23–26^

In this study, we optimized the single enteral bolus method of 1-^13^C-propionate oxidation in healthy volunteers^26,28^ and a large number of MMA subjects, including a subset who received an organ (liver and/or kidney) transplantation. The 1-^13^C-propionate oxidative capacity was correlated with clinical and laboratory markers of disease severity, and fully normalized after liver transplantation. These test characteristics support the use of 1-^13^C-propionate oxidation as both a predictive and pharmacodynamic response biomarker in MMA, and thus highlight the use of this assay to measure restoration of hepatic MMUT function in small molecule, mRNA, gene, and/or cell therapy clinical trials.

## MATERIALS AND METHODS

### Study subjects

Subjects were enrolled via a longitudinal natural history protocol (clinicaltrials.gov identifier: NCT00078078). The specific subtype (*mut*^0^, *mut* ^*−*^, *cblA*, *cblB*) of isolated MMA was assigned based on molecular genetic analysis and, when available, cellular biochemical studies in fibroblasts as previously described.^4,5^

The cohort was comprised of 57 participants: 38 *mut*^0^, 7 *mut* ^*−*^, 10 *cblA*, and 2 *cblB*; 19 were transplant recipients, 17 with *mut*^0^ MMA, 1 with *mut*^*−*^ MMA, and 1 with cblA. Six patients were tested both before and after organ transplantation: three LT/LKT and three KT (Table 1). A total of 83 propionate breath tests were performed in this patient cohort. While the majority of participants were measured once, 12 MMA subjects repeated the study twice and 6 MMA subjects repeated the study three times during separate visits to the National Institutes of Health (NIH) Clinical Research Center (CRC). Study participants were evaluated at steady state. The control arm consisted of eight healthy adult volunteers and eight heterozygote parents; all were screened to rule out B_12_ deficiency prior to enrollment.

**Table 1:** Demographic, molecular, and clinical data of MMA subjects.

#	Age (years)	Sex	Race	Transplant	Gene	Variant 1	Variant 2	Serum MMA (μM)	% 1-^13^C3 dose metabolized (60 and 120 minutes)		Tmax Δ^13^C (minutes)	eGFR (mL/min/1.73 m^2^)
*1*	*4.2*	*M*	*W*	*-*	*MMUT*	*c.1207C>T, p.Arg403Ter*	*Unknown*	*774*	*12.51*	*18.97*	*30*	*110.1*
*2*	*5.4*	*M*	*A*	*-*	*MMUT*	*c.1630_1631delinsTA* *p.Gly544Ter*	*c.1106G>A, p.Arg369His*	*1,465*	*5.70*	*11.82*	*45*	*37.4*
*3*	*8.7*	*M*	*M*	*-*	*MMUT*	*c.682C>T, p.Arg228Ter*	*c.322C>T, p.Arg108Cys*	*2,011*	*6.43*^*c*^	*13.92*^*c*^	*25*	*40.2*
*4*	*8.8*	*M*	*W*	*-*	*MMUT*	*c.1106G>A, p.Arg369His*	*c.1324G>C, p.Ala442Pro*	*4,170*	*11.16*	*25.52*	*60*	*26.1*
*5*	*9.6*	*F*	*B*	*-*	*MMUT*	*c.91C>T, p.Arg31Ter*	*c.250G>T, p.Glu84Ter*	*167*	*12.31*	*20.39*	*30*	*92.4*
*6*	*9.7*	*M*	*W*	*-*	*MMUT*	*c.52C>T, p.Gln18Ter*	*c.1038_1040del, p.Leu347del*	*2,992*	*11.79*	*23.66*	*50*	*35.8*
*7*	*11.2*	*M*	*W*	*-*	*MMUT*	*c.1106G>A, p.Arg369His*	*c.1324G>C, p.Ala442Pro*	*1,702*	*18.69*	*35.35*	*45*	*52.5*
*8*	*11.4*	*F*	*B*	*-*	*MMUT*	*c.682C>T, p.Arg228Ter*	*c.1108A>C, p.Thr370Pro*	*258*	*8.89*	*N/A*	*50*	*118.3*
*9*	*11.7*	*M*	*A*	*-*	*MMUT*	*c.1560G>C, p.Lys520Asn*	*c.1560G>C, p.Lys520Asn*	*596*	*16.07*	*31.14*	*45*	*60.2*
*10*	*11.8*	*M*	*W*	*-*	*MMUT*	*c.372_374dup p.Lys124_Asp125insGlu*	*c.146_147ins279*^*d*^	*1,407*	*6.74*	*18.59*	*90*	*46.4*
*11*	*13.6*	*F*	*H*	*-*	*MMUT*	*c.1196_1197del p.Val399GlufsTer24*	*c.1106G>A, p.Arg369His*	*1,530*	*16.78*	*35.65*	*60*	*40.8*
*12*	*13.7*	*M*	*W*	*-*	*MMUT*	*c.1741C>T, p.Arg581Ter*	*c.753* *+* *2T>A, p.splice*	*3,127*	0.63^**c**^	7.20^**c**^	*90*	*51.0*
*13*	*14.2*	*F*	*W*	*-*	*MMUT*	*c.1924G>C, p.Gly642Arg*	*c.1924G>C, p.Gly642Arg*	*3,092*	*14.19*^*c*^	*31.25*^*c*^	*45*	*31.9*
*14*	*14.4*	*F*	*A*	*-*	*MMUT*	*c.682C>T, p.Arg228Ter*	*c.785G>T, p.Ser262Ile*	*1,399*	*5.11*	*N/A*	*60*	*54.0*
*15*	*15.1*	*F*	*W*	*-*	*MMUT*	*c.91C>T, p.Arg31Ter*	*c.2053_2055dup* *p.Leu685dup*	*341*	*21.38*^*c*^	*41.37*^*c*^	*45*	*51.1*
*16*	*15.6*	*F*	*A*	*-*	*MMUT*	*c.2179C>T, p.Arg727Ter*	*c.2179C>T, p. Arg727Ter*	*2,147*	*6.21*	*16.81*	*60*	*31.9*
*17*	*15.7*	*F*	*W*	*-*	*MMUT*	*c.682C>T, p.Arg228Ter*	*c.607G>A, p.Gly203Arg*	*551*	*8.52*^*c*^	*20.24*^*c*^	*40*	*54.0*
*18*	*16.5*	*F*	*B*	*-*	*MMUT*	*c.281G>T, p.Gly94Val*	*c.1108A>C, p.Thr370Pro*	*234*	*19.85*	*34.55*	*30*	*36.9*
*19*	*16.8*	*F*	*A*	*-*	*MMUT*	*c.2078del, p.Gly693AspfsTer12*	*c.2078del, p.Gly693AspfsTer12*	*1,239*	*11.80*	*N/A*	*45*	*91.4*
*20*	*18.0*	*F*	*W*	*-*	*MMUT*	*c.323G>A, p.Arg108His*	*c.1867G>C, p.Gly623Arg*	*400*	*6.38*	*N/A*	*60*	*58.3*
*21*	*18.8*	*F*	*A*	*-*	*MMUT*	*c.643G>T, p.Gly215Cys*	*c.643G>T, p. Gly215Cys*	*1,094*	*16.35*	*29.96*	*30*	*47.8*
*22*	*19.5*	*F*	*W*	*-*	*MMUT*	*c.29dup, p.Leu10PhefsTer39*	*c.1658del, p.Val553GlyfsTer17*	*2,260*	*6.12*^*c*^	*15.09*^*c*^	*60*	*70.3*
*23*	*23.7*	*M*	*B*	*-*	*MMUT*	*c.1207C>T, p.Arg403Ter*	*c.1360G>A, p.Gly454Arg*	*730*	*14.24*	*27.01*	*45*	*103.9*
*24*	*25.1*	*M*	*W*	*-*	*MMUT*	*c.572C>A, p.Ala191Glu*	*c.655A>T, p.Asn219Tyr*	*2,123*	*6.19*	*15.81*	*60*	*38.5*
*25*	*28.1*	*F*	*W*	*-*	*MMUT*	*c.623_624del, p.Val208AlafsTer2*	*c.322C>T, p.Arg108Cys*	*1,157*	*14.29*	*25.94*	*52*	*33.1*
*26*	*33.7*	*M*	*W*	*-*	*MMUT*	*c.753* *+* *2T>A, p.splice*	*c.1560* *+* *1G>T, p.splice*	*4,754*	*7.02*	*16.13*	*60*	*45.8*
**27**	**3.4**	**M**	**W**	**-**	***MMUT***	**c.372_374dup, p.Lys124_Asp125insGlu**	**c.842T>C, p.Leu281Ser**	**36**	**39.46**	**48.23**	**15**	**76.0**
**28**	**4.8**	**M**	**B**	**-**	***MMUT***	**c.91C>T, p.Arg31Ter**	**c.2150G>T, p.Gly717Val**	**19.5**	**18.42**	**26.93**	**25**	**22.3**
**29**	**5.9**	**M**	**B**	**-**	***MMUT***	**c.88C>T, p.Gln30Ter**	**c.299A>G, p.Tyr100Cys**	**47**	**38.81**	**50.65**	**30**	**84.2**
**30**	**8.5**	**F**	**B**	**-**	***MMUT***	**c.88C>T, p.Gln30Ter**	**c.299A>G, p.Tyr100Cys**	**266**	**28.89**	**40.86**	**33**	**108.2**
**31**	**9.2**	**M**	**W**	**-**	***MMUT***	**c.1181T>A, p.Leu394Ter**	**c.1924G>C, p.Gly642Arg**	**37**	**37.70**^**c**^	**48.35**^**c**^	**20**	**109.6**
**32**	**9.6**	**F**	**B**		***MMUT***	**c.2150G>T, p. Gly717Val**	**c.281G>T, p.Gly94Val**	**168**	**28.47**	**48.82**	**30**	**91.9**
33	4.2	M	W	-	*MMAA*	c.433C>T, p.Arg145Ter	c.433C>T, p.Arg145Ter	80	35.03	43.09	15	87.7
34	9.9	M	M	-	*MMAA*	c.387C>A, p.Tyr129Ter	c.593_596del, p.Thr198SerfsTer6	27	35.10^**c**^	51.79^**c**^	30	116.6
35	10.8	M	W	-	*MMAA*	c.433C>T, p.Arg145Ter	c.1076G>A, p.Arg359Gln	36	34.90	52.86	15	101.9
36	13.7	M	W	-	*MMAA*	c.433C>T, p.Arg145Ter	c.1075C>T, p.Arg359Ter	18.3	38.33^c^	54.39^c^	15	114.7
37	19.1	M	W	-	*MMAA*	c.433C>T, p.Arg145Ter	c.433C>T, Arg145Ter	25	30.99	46.93	25	76.5
38	26.5	M	W	-	*MMAA*	c.433C>T, p.Arg145Ter	c.593_596del, p.Thr198SerfsTer6	27	43.48	62.13	15	83.5
39	27.6	M	W	-	*MMAA*	c.440G>A, p.Gly147Glu	c.450dup, p.Pro151AlafsTer19	50	28.38^c^	40.65^c^	30	78.5
40	41.8	M	NAm	-	*MMAA*	c.433C>T, p.Arg145Ter	c.433C>T, p.Arg145Ter	29	34.04^c^	52.46^c^	25	97.4
41	47.2	M	W	-	*MMAA*	c.433C>T, p.Arg145Ter	c.433C>T, p.Arg145Ter	56	31.81	47.74	30	85.5
***42***	***3.7***	***M***	***W***	***-***	***MMAB***	***c.556C>T, p.Arg186Trp***	***c.556C>T, p.Arg186Trp***	***805***	***8.35***	***16.34***	***40***	***36.1***
***43***	***34.1***	***M***	***W***	***-***	***MMAB***	***c.700C>T, p.Gln234Ter***	***c.556C>T, p.Arg186Trp***	***279***	***32.75***	***47.22***	***17***	***39.3***
*44*	*17.2*	*F*	*W*	*KT*	*MMUT*	*c.927G>A, p.Trp309Ter*	*c.983T>C p.Leu328Pro*	*2,477*	*9.41*	*21.1*	*60*	*23.4*
*45*	*18.1*	*F*	*W*	*KT*^*a*^	*MMUT*	*c.1924G>C, p.Gly642Arg*	*c.1924G>C, p.Gly642Arg*	*688*	*13.42*	*28.75*	*60*	*58.9*
*46*	*32.2*	*M*	*A*	*KT*^*b*^	*MMUT*	*c.1741C>T, p.Arg581Ter*	*c.1741C>T, p.Arg581Ter*	*445*	*12.30*	*24.49*	*30*	*70.8*
*47*	*37.5*	*M*	*W*	*KT*^*a*^	*MMUT*	*c.753* *+* *2T>A, p.splice*	*c.1560* *+* *1G>T, p.splice*	*11,991*	*10.21*	*21.17*	*60*	*19.4*
**48**	**30.3**	**M**	**M**	**KT**	***MMUT***	**c.1760A>C, p.Tyr587Ser**	**c.2150G>T, p.Gly717Val**	**263**	**25.15**	**36.43**	**25**	**78.9**
49	27.1	F	W	KT	*MMAA*	c.433C>T, p.Arg145Ter	c.586C>T, p.Arg196Ter	24	23.74	35.35	15	74.4
*50*	*9.3*	*F*	*W*	*LKT*	*MMUT*	*c.682C>T, p.Arg228Ter*	*c.1287C>G, p.Tyr429Ter*	*231*	*42.12*	*60.83*	*30*	*75.6*
*51*	*10.7*	*M*	*M*	*LKT*^*a*^	*MMUT*	*c.682C>T, p.Arg228Ter*	*c.322C>T, p.Arg108Cys*	*121*	*34.31*	*45.76*	*20*	*77.7*
*52*	*11.7*	*F*	*W*	*LKT*	*MMUT*	*c.878A>C, p.Gln293Pro*	*c.878A>C, p.Gln293Pro*	*154*	*36.52*	*47.50*	*10*	*44.9*
*53*	*13.0*	*M*	*A*	*LKT*	*MMUT*	*c.2179C>T, p.Arg727Ter*	*c.2179C>T, p.Arg727Ter*	*96*	*31.80*	*42.74*	*15*	*80.9*
*54*	*13.5*	*M*	*W*	*LKT*	*MMUT*	*c.682C>T, p.Arg228Ter*	*c.670G>T, p.Glu224Ter*	*385*	*27.88*	*48.78*	*20*	*50.7*
*55*	*15.7*	*M*	*M*	*LKT*	*MMUT*	*c.349G>T, p.Glu117Ter*	*c.1038_1040del p.Leu347del*	*57*	*27.69*	*N/A*	*15*	*36.1*
*56*	*16.8*	*M*	*W*	*LT*^*a*^	*MMUT*	*c.1741C>T, p.Arg581Ter*	*c.753* *+* *2T>A, p.splice*	*402*	*28.59*	*48.68*	*45*	*66.5*
*57*	*18.5*	*M*	*W*	*LKT*	*MMUT*	*c.1207C>T, p.Arg403Ter*	*c.1105C>T, p.Arg369Cys*	*395*	*32.49*	*44.59*	*7*	*59.4*
*58*	*19.1*	*M*	*A*	*LT*	*MMUT*	*c.1106G>A, p.Arg369His*	*c.1106G>A, p.Arg369His*	*1,021*	*42.45*	*54.41*	*10*	*33.2*
*59*	*23.9*	*F*	*W*	*LKT*^*a*^	*MMUT*	*c.29dup, p.Leu10PhefsTer38*	*c.1658del, p.Val553GlyfsTer17*	*9.5*	*35.65*	*49.43*	*15*	*69.8*
*60*	*30.4*	*F*	*W*	*pLKT*	*MMUT*	*c.1106G>A, p.Arg369His*	*c.1106G>A, p.Arg369His*	*2,246*	*13.06*	*24.94*	*45*	*49.5*
*61*	*31.1*	*F*	*W*	*LKT*	*MMUT*	*c.2053_2055dup p.Leu685dup*	*c.2053_2055dup p.Leu685dup*	*382*	*27.88*	*39.92*	*15*	*95.5*
*62*	*37.2*	*F*	*H*	*LKT*	*MMUT*	*c.826G>T, p.Glu276Ter*	*c.1106G>A, p.Arg369His*	*255*	*24.88*	*36.57*	*20*	*49.8*

Age at the time of stable isotope breath testing, as well as the molecular genetic analysis of the *MMUT*, *MMAA*, and *MMAB* genes and selected variables of the study are provided for each of the MMA subjects. Italics for mut^0^, bold for mut^–^, bold and italics for cblB (MMAB) individuals and underline for cblA (MMAA). Serum MMA concentration is provided in micromole/liter, μmol/L or μM (normal levels <0.4 μmol/L).

*A* Asian, *B* Black, *eGFR* estimated glomerular filtration rate, *H* Hispanic, *KT* kidney transplant, *LT* liver transplant, *LKT* combined liver–kidney transplant, *M* Mixed race, *MMA* methylmalonic acid, *N/A* data not available, *W* White.

^a^Subjects tested before and after liver and/or kidney transplantation.

^b^Repeat study after a second KT.

^c^Designates subjects where in vitro ^14^C-propionate fibroblast enzymatic assays were also available, provided in detail in Supplemental Table 1.

^d^Individual 10 has a large 279- bp insertion comprised of an 183- bp segment that aligns to the 3’UTR of the *ENO1* gene on chromosome 1 (8861004_8861186) and a ~96- bp polyA repeat that result in no detectable messenger RNA (mRNA) (PMID: 27233228).

Clinical data derived from the natural history protocol were analyzed per disease subtype and transplant status and are summarized in (Table 2). This included anthropometric measurements using the package Epi Info^TM^, version 3.5.1. (Centers for Disease Control and Prevention, Atlanta, GA, USA). Dual energy X-ray absorptiometry (DXA, Hologic Delphi A; Hologic, Bedford, MA, USA) was employed for bone mineral density (BMD). Neurocognitive assessments were performed using standardized instruments for the patient’s age and functional status, as described previously.^29^ In young children (*n* = 3) or subjects with marked sensory, motoric, or cognitive impairment (*n* = 4), neurocognitive functioning was assessed using the Vineland Adaptive Behavior Scales (this comprised only 7/51 [14%] of patients tested and was therefore combined with full-scale intellectual quotient [FSIQ] for the statistical analysis). Dietary information (kcal/day, complete/deficient protein g/kg/day), and biochemical parameters including plasma quantitative amino acids, acylcarnitine profiles, plasma and urine methylmalonic acid levels, renal (cystatin C, estimated glomerular filtration rate [eGFR] using age-appropriate creatinine and/or cystatin C-based CKiD equations, https://www.kidney.org), and mitochondrial function biomarkers (fibroblast growth factor 21, FGF21 and growth differentiation factor 15, GDF15) were assessed at each visit.^15,16,30,31^

**Table 2:** Descriptive statistics of clinical data per disease subtype and transplant status.

Variable	MMA subtype/gene name				*mut*^0^ subjects transplant status	
	*mut*^0^*/MMUT*	*mut*^*−*^/*MMUT*	*cblA/MMAA*	*cblB/MMAB*	Liver ± kidney	Kidney
*N*	26	6	9	2	13	4
Males	12	4	9	2	7	2
Females	14	2	0	0	6	2
Age	15.2 ± 6.7	6.9 ± 2.5	22.3 ± 14.7	3.7/34.1	19.3 ± 8.7	26.3 ± 10.1
% 1-^13^C3 metabolized 60 minutes	10.97 ± 5.2 7	31.95 ± 8.25^b^	34.67 ± 4.37^c^	8.35/32.75	31.17 ± 7.72^c^	11.33 ± 1.84
120 minutes	23.47 ± 8.99	44.10 ± 8.95^b^	50.26 ± 6.45^c^	16.34/47.22	45.34 ± 9.02^c^	23.86 ± 3.61
AUC dose metabolized 60 minutes	257.12 ± 123.6	944.11 ± 303.47^a^	1,000.40 ± 172.28^c^	191.2/1040	917.01 ± 248.56^c^	249.40 ± 50.0
120 minutes	1,322.9 ± 568.14	3,289.66 ± 937.84^a^	3,645.22 ± 477.60^c^	988.20/3,485.0	3,222.4 ± 730.66^c^	1,337.50 ± 210.34
Tmax Δ^13^C (min)	50.52 ± 16.42	25.50 ± 6.89^c^	22.22 ± 7.12^c^	40.0/17.0	20.53 ± 12.29^c^	52.50 ± 15.0
REE (cal)	1,182.84 ± 261.3	816.16 ± 370.96	1,373.44 ± 278.46	609.3/1,653.1	1,351.12 ± 312.62	1,319.13 ± 181.12
REE (cal)/FFM (kg)	49.72 ± 10.48	51.63 ± 8.67	36.71 ± 7.17	na/31.43	41.73 ± 8.69	54.1
Height *z*-score	−1.86 ± 1.74	−1.31 ± 1.34	−0.39 ± 0.98	−1.94/−1.31	−1.59 ± 0.84	−2.58 ± 1.56
BMI *z*-score	0.45 ± 1.22	1.03 ± 1.35	−0.51 ± 1.21	1.94/1.67	0.71 ± 1.23	−0.15 ± 1.95
OFC *z*-score	−0.58 ± 1.46	−0.02 ± 1.47	0.67 ± 1.28	−0.15/0.50	−0.33 ± 1.47	−0.76 ± 1.81
BMD *z*-score: subtotal	−2.65 ± 1.48	−1.71 ± 0.77	−0.62 ± 1.29	na/−0.50	−1.87 ± 1.30	−4.19
BMD *z*-score: height corrected, subtotal	−1.27 ± 0.92	−1.01 ± 0.56	0.23 ± 1.71	na/1.09	−0.93 ± 1.22	−1.67
Complete protein intake (%RDA)	80.33 ± 30.45	84.52 ± 14.39	102.43 ± 26.6	49.08/90.91	93.28 ± 33.15	74.87 ± 20.80
FSIQ/ABC	70.6 ± 22.33	89.7 ± 14.88	92.1 ± 14.22^a^	65/na	69.84 ± 19.68	87.50 ± 22.54
eGFR creatinine (ml/min/1.73 m^2^)	63.83 ± 34.38	126.22 ± 35.94^b^	108.19 ± 27.66^b^	45.96/37.59	65.95 ± 27.93	43.83 ± 26.95
eGFR creat/cyst-C (ml/min/1.73 m^2^)	56.65 ± 26.59	97.09 ± 11.25^c^	90.76 ± 13.01^c^	39.33/36.19	60.78 ± 18.70	43.18 ± 25.57
Serum MMA (μmol/L)	1,604.61 ± 1,220.02	95.59 ± 99.40^b^	39.26 ± 21.04^c^	805.0/279.0	459.53 ± 589.85^b^	3,900.25 ± 5,469.40
Acylcarnitine/free carnitine ratio	3.22 ± 2.27	0.79 ± 0.63^b^	0.32 ± 0.14^c^	2.93 − 0.7	0.92 ± 0.36^c^	2.15 ± 1.6
FGF21 (pg/ml)	5,817.21 ± 7,410.63	1,167.14 ± 1,431.66	319.84 ± 229.31^a^	19,791.8 − 400.4	1,881.11 ± 2,485.01^a^	5,739.38 ± 5,313.32
GDF15 (pg/ml)	3,019.07 ± 2,182.89	351.81 ± 199.42	290.25 ± 177.68	na − 1,631.5	1,767.41 ± 1,299.28	2,603.72 ± 2,187.10

A *p* value of multiple comparisons for FGF21 was <0.05 for the log10 transformed variable, not the absolute values. One-way analysis of variance (ANOVA) and post hoc correction for multiple comparisons by Bonferroni or Tamhane was used depending on the result of Levine’s test of homogeneity of variances, between MMA subtypes and muto and within mut^0^ subtype, between individuals without and with an organ transplant.

*AUC* area under the curve, *BMD* bone mineral density measured by dual energy X-ray absorptiometry (DXA) (subtotal refers to BMD of total body minus head), *BMI* body mass index, *eGFR* estimated glomerular filtration rate (based on creatinine or combined creatinine and cystatin C values, per the Bedside Schwartz and CKiD 2012 equations), *FGF21* fibroblast growth factor 21, *FFM* fat-free or lean mass (in kilograms), *FSIQ* full-scale IQ (by age-appropriate cognitive assessment, or ABC score based on Vineland adaptive behavior scales), *GDF15* growth differentiation factor 15, *MMA* methylmalonic acid, *OFC* occipitofrontal (head) circumference, *RDA* recommended daily allowance, *REE* resting energy expenditure (in calories), *Tmax* time to reach maximum delta of exhaled ^13^CO_2_/^12^CO_2_.

^a^*P* <  0.05.

^b^*P* <  0.01.

^c^*P* < 0.001.

### Measurement of resting CO_2_ production rate

We utilized an open-circuit indirect calorimetry method (ventilated hood) for measuring O_2_ consumption (VO_2_) and CO_2_ production (VCO_2_) typically between 7 a.m. and 8:00 a.m. with the child resting supine in bed for a minimum of 30 minutes.^32^ We calculated resting energy expenditure (REE in kcal/day) using the modified Weir equation [3.9(VO_2_) + 1.1(VCO_2_)].^33^ Subjects were tested after 12 hours of fasting if tolerated, or after 3 hours for patients on continuous feedings overnight. In five subjects who were not compliant or could not maintain a resting state due to a movement disorder, a predicted VCO_2_ rate was calculated using the formula: 300 mmol h^−1^ m^−2^ body surface area.^34^

### Stable isotope administration, breath collection, and analysis

Sodium 1-^13^C-propionate (CH_3_CH_2_^13^COO^-^ Na^+^, MW: 97.05 g/mol, 99 atom% ^13^C, #CLM-771-MPT from Cambridge Isotope Laboratories, Andover, MA, USA) was used for the studies. A dose of 0.5 mg/kg body weight was prepared at a concentration of 1 mg/mL with sterile water the morning of testing by the NIH pharmacy and administered orally or through a gastrostomy tube (*n* = 13/59, 22%) over 2 minutes. No side effects occurred with the administration of label by either route. Breath samples were collected via disposable breath collection kits (EasySampler™ Breath Test Kit, Quintron) prior to isotope administration, and at specified time points over 2 hours. The isotopic ratio (^13^C/^12^C) in expired CO_2_ was determined by isotope ratio mass spectrometry (Metabolic Solutions, Nashua, NH, USA). Results were reported as follows: δ^13^C (per mil,%_0_) = [(^13^C:^12^C_sample_/ ^13^C:^12^C_standard_) - 1] * 1,000. Percent dose oxidized = VCO_2_ × Σ (APE/mmol ^13^C administered) * 100, where APE stands for atomic percent excess, the level of isotopic abundance above a given background reading, which is considered zero. The proposed fate of the heavy ^13^C carbon atom of the 1-^13^C-propionate administered to exhaled ^13^CO_2_ is provided in Supplemental Fig. 1. A second method, utilizing the BreathID® Exalenz device, was also employed. Exalenz BreathID® is cleared by the FDA for use with ^13^C-urea for the diagnosis of *H. pylori* infection.^35–38^ Further details of the methods are provided in Supplemental Fig. 2.

### Statistical analysis

Statistical analysis was performed using IBM® SPSS Statistics version 21 (Chicago, IL, USA) or GraphPad Prism version 7.0c (Carey, NC, USA) software. Pearson and Spearman rank correlation coefficient and linear regression were used to evaluate bivariate correlations of propionate oxidation result with clinical parameters. Continuous variables between participants were evaluated with independent Student’s *t*-test or Mann–Whitney *U*-test, one-way analysis of variance (ANOVA), or Kruskal–Wallis ANOVA for normally distributed and nonparametric variables, respectively. Log transformation (decimal) was employed for skewed continuous variables. One-way ANOVA comparisons were performed between MMA subtypes and within the *mut*^0^ individuals before and after LT/LKT or KT. Levine’s test of homogeneity of variances was applied and post hoc correction for multiple comparisons was employed accordingly (Tukey HSD for equal variances, and Tamhane or Dunnett T3 for unequal variances) to calculate individual *P* values between each of the different groups versus *mut*^0^ and between *mut*^0^ nontransplanted and LT/LKT and KT recipients. Results are presented as mean ± SD. *P* value of less than 0.05 was considered statistically significant; levels of significance are denoted by asterisks in figures as follows: *<0.05, **<0.01, ***<0.001, ****<0.0001.

## RESULTS

### Method development and reproducibility in healthy volunteers vs. patients

**Fig. 1:**
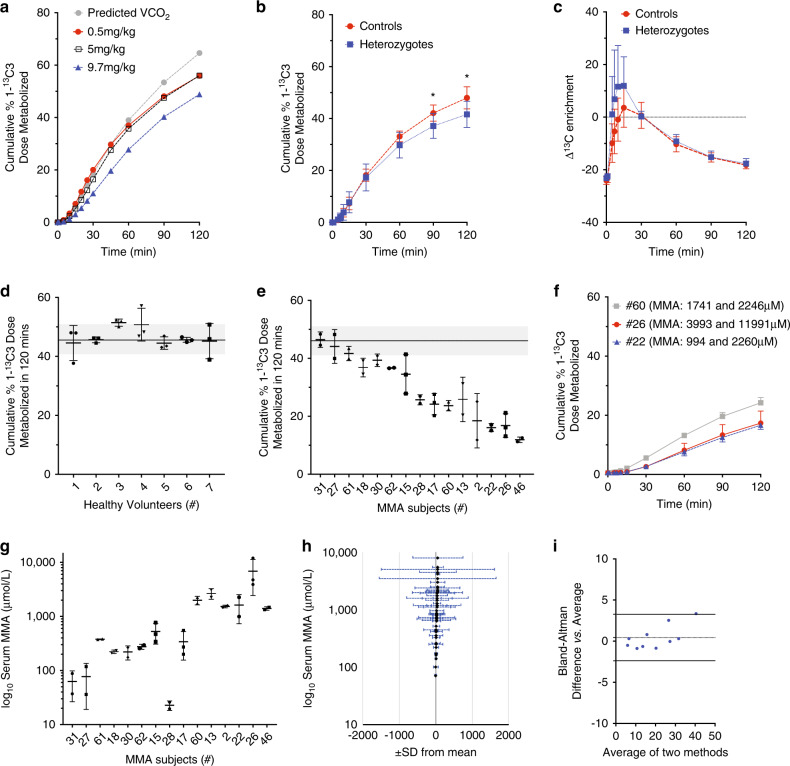
Method development, reproducibility, and test performance. (**a**) Studies in a single healthy adult volunteer showed similar test performance using 2- and 20-fold reduced isotopomer dose. The amount of the administered dose of 1-^13^C-sodium propionate oxidized after 2 hours was 48.8% with the 9.7 mg/kg dose (blue line), 56.03% with a 5 mg/kg, and 55.91% with a 0.5 mg/kg dose (red line). When predicted CO_2_ rather than the VCO_2_ measured by the metabolic cart was used for calculations with the 0.5 mg/kg dose, result were identical up to the 60-minute timepoint. (**b**) Healthy adult controls (*n* = 8, red) and heterozygote parents (*n* = 8, blue) volunteered for the study. At 120 minutes, controls metabolized 47.9 ± 4.26% and heterozygotes 41.6 ± 5.05% of the administered 1-^13^C-propionate dose (*P* = 0.01). (**c**) Enrichment of ^13^CO_2_/^12^CO_2_ in the exhaled air collected in frequent intervals in the first 15 minutes of the study showed a similarly rapid propionate oxidation in both, controls and heterozygote subjects. (**d**) Healthy adult volunteers (*n* = 7, labeled 1–7) repeated the study three times each (scatter dot plot, mean ± SD for each participant). (**e**) Cumulative dose oxidized at 120 minutes is depicted for repeat 1-^13^C-propionate breath tests in 15 subjects with *mut* subtype methylmalonic acidemia (MMA) (scatter dot plot, mean ± SD). Patient numbers (#) correlate to the IDs in Table 1 and are ranked from high to low 1-^13^C-propionate oxidation capacity. Gray-shaded band in (**d**, **e**) corresponds to the mean ± SD of healthy controls. (**f**) 1-^13^C-propionate breath test showed near identical propionate oxidation in selected subjects with significantly different serum methylmalonic acid concentrations at the time of testing. (**g**) Serum methylmalonic acid concentrations (μmol/L, mean ± SD is shown for each subject) corresponding to the subjects depicted in (**e**). Patients with higher oxidative capacity had lower serum methylmalonic acid concentrations. (**h**) Serum methylmalonic acid means (*y*-axis) and standard deviations (*x*-axis) are depicted for 57 subjects with MMA, who had more than one methylmalonic acid measurement during their week-long stay at the National Institutes of Health (NIH) Clinical Research Center (CRC). (**i**) A Bland–Altman plot shows near perfect agreement between the two methods employed for measuring the delta over baseline ^13^CO_2_/^12^CO_2_ enrichment: isotope ratio mass spectrometry (IRMS) and the Exalenz BreathID® device. The plot depicts the differences between the two techniques on the *y*-axis against the average of the two methods on the *x-*axis (****P < 0.0001. ***P < 0.001, **P < 0.01, *P < 0.05).

Dose-finding studies were performed first in a single healthy volunteer. Three doses of 1-^13^C-propionate were tested: 100 µmol or 9.7 mg/kg body weight (BW) as described in Barshop et al.,^26^ an intermediate dose of 5 mg/kg, and a low dose of 0.5 mg/kg, as used in recent studies aimed at detecting B_12_ deficiency.^28^ The result were identical between the 5 and 0.5 mg/kg dose (Fig. 1a) and the lower dose was chosen for further study to minimize propionate exposure in MMA subjects.

Conversion of labeled propionate to ^13^CO_2_ was rapid in healthy volunteers with 47.9 ± 4.2% of the label oxidized in 120 minutes (Fig. 1b), and an average time of maximum enrichment at 22.5 ± 12.9 minutes (Fig. 1c). Heterozygote parents showed a similar time of maximum enrichment and a small decrease in activity/enrichment at the 90- and 120-minute time points (*P* = 0.02 and 0.01, respectively) compared with controls; of note, the average age of the healthy volunteers was lower than that of the parents (25.1 ± 4.3 vs. 45.5 ± 9.5 years, *P* = 0.0001). To evaluate inter- and intraindividual variability, seven healthy volunteers repeated the study three times, with 1- to 3-week intervals between repeat testing (Fig. 1d). At 120 minutes, the coefficient of variation (CV) for dose metabolized within each subject ranged from 1.83% to 13.33%, with an average of 6.94 ± 5.3%, while the CVs between the seven subjects was 11.9%, 9.6%, and 5.6% for each of the three sets of replicate studies performed.

Variation between testing was higher in subjects with MMA, who had repeat testing at variable intervals ranging from 2 months to 4 years between their follow-up NIH visits. At 120 minutes, the CV for 1-^13^C-propionate dose metabolized in 15 *mut* patients, who had repeated breath testing 2–3 times each, ranged from 0.39% to 50.84% with an average of 14.02 ± 12.86% (Fig. 1e). The higher variations occurred in subjects with changing renal status between testing (eGFR decreased from 57 to 43 mL/kg/1.73 m^2^, subject 15), variable VCO_2_ measurements (146.0 to 159.2 ml/min, subject 17), or possibly related to incomplete dose administration or other factors (subject 2). Notably, acceptable 1-^13^C-propionate breath test CV of <15% was observed over a wide range of enzymatic activity and despite significantly different serum methylmalonic acid concentrations (Fig. 1f). In contrast, CV for serum methylmalonic acid concentrations in the same subjects undergoing repeat testing was more variable, ranging from 1.5% to 75.3% with an average of 31.38 ± 24.55% (Fig. 1g). Serum methylmalonic acid values drawn during the same week-long patient stay in the NIH CRC showed a wide variation (Fig. 1h), and were significantly affected by renal dysfunction (when serum methylmalonic acid >1,000 μmol/L), as described previously.^14,15,30^

Next, we compared the isotope ratio mass spectrometry (IRMS) result with those simultaneously measured with the BreathID® in nine MMA subjects. The correlation coefficient was *r* = 0.996 (*P* < 0.0001), while the Bland–Altman plot showed a 95% limits of agreement ranging between -2.386% and 3.266% between the two methods (Fig. 1i).

### 1-^13^C-propionate breath testing reflects the severity of MMA subtypes and is restored after liver transplantation

**Fig. 2:**
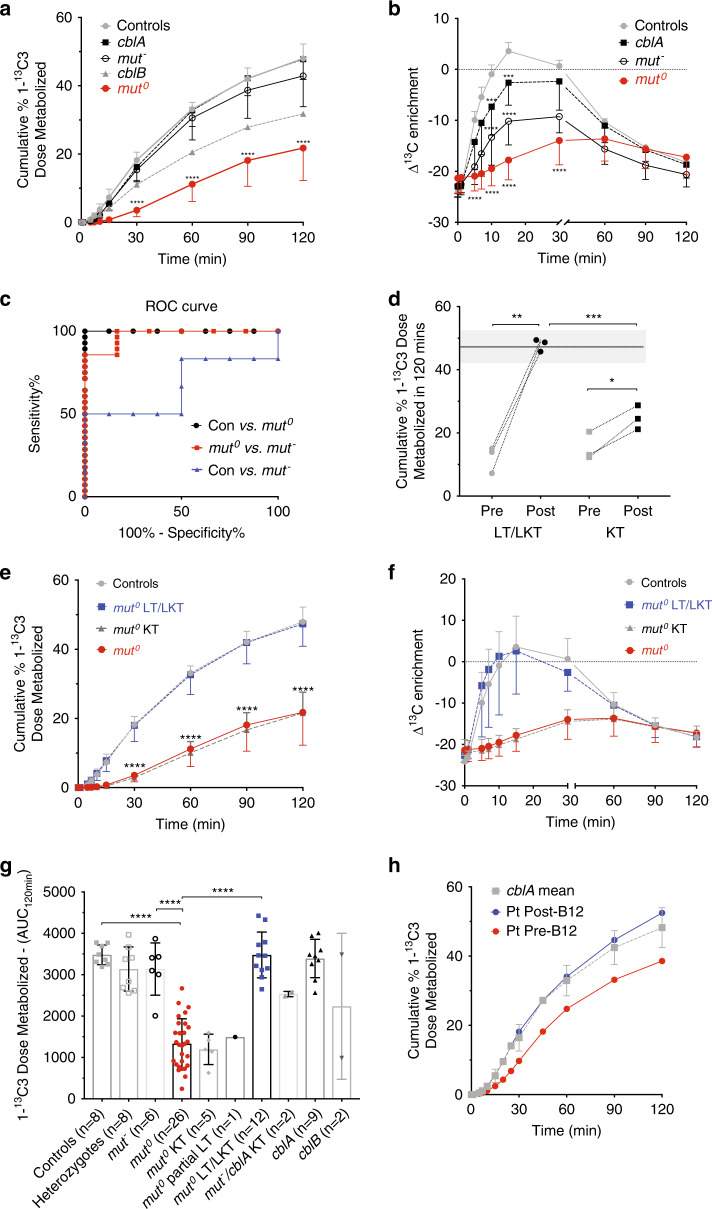
1-^13^C-propionate oxidation in methylmalonic acidemia (MMA) patients: correlations with subtype severity and response after organ transplantation. (**a**) The cumulative percent dose of administered sodium 1-^13^C-propionate metabolized by 120 minutes is depicted for controls (*n* = 8) and MMA subjects, per disease subtype (*mut*^*0*^ *n* = 26, *mut*^–^ *n* = 6, *cblB n* = 2, *cblA n* = 9). The severe *mut*^0^ patients (marked in red) had a range of oxidative capacity with a mean ± SD of 21.77 ± 9.50% at 120 minutes (*P* < 0.0001 compared with the controls 47.96 ± 4.26% and *cblA* 47.75 ± 5.91%; *P* = 0.0002 compared with *mut*^–^ individuals, 42.88 ± 9.02%, and *P* = NS to *cblB* 31.78 ± 21.82%). (**b**) The delta over baseline ^13^C enrichment in exhaled breath samples is depicted in controls and MMA subjects per subtype. A slower time to maximum enrichment was observed in *mut*^0^ subjects, 46.59 ± 18.25 minutes, as opposed to 22.5 ± 11.81 in controls, 23.18 ± 6.8 in *cblA* patients, and 32.50 ± 11.65 in *mut*^–^. Asterisks correspond to *P* values compared with controls for each time point. (**c**) A receiver operating curve (ROC) is depicted for the cumulative dose oxidized in 120 minutes between controls and *mut*^*0*^ (area under the curve [AUC]= 1, *P* < 0.0001) as well as between *mut*^*0*^ and *mut*^–^ (AUC: 0.9345 ± 0.0655, *P* = 0.001). Method sensitivity between controls and *mut*^–^ patients was low (AUC: 0.687, *P* = NS). (**d**) Three patients were tested before and after combined liver/kidney transplant (LT/LKT), and three before and after kidney transplant (KT). One subject was tested before and after a second KT procedure after failure of the first kidney graft. On average pre- and post-transplant values were significant even for the kidney transplant recipients. (**e**, **f**) Cumulative percent dose metabolized and enrichment curves are depicted for *mut*^0^ patients without and with a LT/LKT transplant (marked in red and blue, respectively) or isolated KT procedure. LT/LKT but not KT resulted in complete restoration of propionate oxidation to control values. (**g**) 1-^13^C-propionate oxidation breath test result in controls and MMA patients per subtype and transplant status are depicted as areas under the curve, for patients with more than one test, the average value is represented (scatter plot of individual values, boxes and error bars represent mean ± SD). (**h**) An adult with *cblA* defect unable to comply with his hydroxocobalamin intramuscular injections was tested before and 2 months after B_12_ therapy, showing improved oxidation. (****P < 0.0001. ***P < 0.001, **P < 0.01, *P < 0.05).

Subjects with *MMUT* variants known to be associated with higher enzymatic activity and protein expression (*mut*^−^ subtype), as well as the B_12_-responsive subtype of cobalamin A deficiency (*MMAA*), exhibited ^13^C-isotopomer oxidative capacity similar to the healthy control mean of 47.96 ± 4.26% cumulative 1-^13^C-propionate dose metabolized at 120 minutes (*P* = NS vs. control values, Fig. 2a); *mut*^0^ patients showed a range of enzymatic activity with a mean of 21.77 ± 9.50% (*P* < 0.0001 compared with controls and *cblA*; *P* = 0.0002 compared with *mut*^−^ individuals). The reduced recovery of ^13^C-isotopomer dose in exhaled breath CO_2_ in *mut*^0^ subjects was also accompanied by a significantly delayed time to maximum enrichment of 46.59 ± 18.25 minutes (*P* = 0.0046) compared with 22.5 ± 11.81 minutes in controls (Fig. 2b). The receiver operating curve (ROC) for the cumulative 1-^13^C-propionate dose oxidized in 120 minutes to distinguish between the *mut*^0^ MMA subtype from healthy controls was excellent (area under the curve [AUC]= 1, *P* < 0.0001), as well as for *cblA* (AUC: 0.933 ± 0.039, *P* < 0.0001) and *mut*^−^ (AUC: 0.934 ± 0.063, *P* = 0.001), but it could not differentiate between control and *cblA* or *mut*^−^ *(*AUC: 0.687, *P* = NS*)*, suggesting low sensitivity to detect milder enzymatic defects (Fig. 2c). On the other hand, the ROC for the Δ13C enrichment was significant for *mut*^0^ and *mut*^*−*^ (*P* < 0.0001), borderline for *cblA* (*P* = 0.052), and not significant for heterozygotes compared with controls (data not shown). It is unclear whether the sensitivity would improve with testing a higher number of subjects.

To examine the effect of transplants on whole-body propionate oxidation, we next studied a number of MMA patients with a variety of organ transplantation procedures, orthotopic liver (LT), partial liver and kidney (pLKT), combined liver/kidney (LKT) or isolated kidney transplant (KT). We measured 1-^13^C-propionate oxidation before and after organ transplantation in six patients, which showed significant improvement in LT/LKT and a lower magnitude change in isolated KT recipients (Fig. 2d), suggesting the test was sensitive to changes at the individual patient level. A complete restoration of oxidation rates and time to maximum enrichment to control levels was observed in liver or combined liver/kidney transplant recipients, but not isolated kidney recipients (Fig. 2e–g). *cblA* or *mut*^−^ subjects with a KT similarly showed levels close to untransplanted patients with similar genotypes. Minimal oxidation was observed in a *mut*^0^ patient, 21 years following an auxiliary liver allograft along with a kidney transplant complicated by stage 3 chronic kidney disease (CKD). This patient later expired and the autopsy revealed a nonfunctioning liver allograft with significant cirrhosis and vascular changes of chronic rejection (data not shown), explaining the lack of activity compared with the other orthotopic LT recipients (Fig. 2g).

Lastly, we assessed a study participant with *cblA* MMA, who was noncompliant with B_12_ injections, at baseline and 2 months after twice weekly 25 mg/ml B_12_ injections. We documented an increase in the cumulative 1-^13^C-propionate dose metabolized over 120 minutes, accompanied by a decrease of the serum methylmalonic acid concentration from 68 μmol/L to 14.84 μmol/L (normal <0.40 μmol/L) (Fig. 2h).

### 1-^13^C-propionate breath test correlates with clinical outcomes and biomarkers in the MMA cohort

**Fig. 3:**
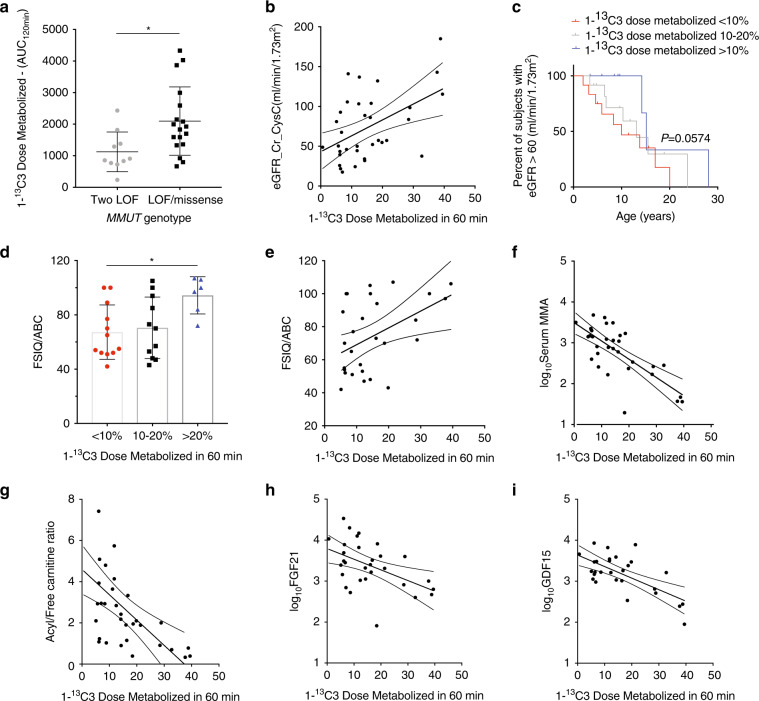
1-^13^C-propionate oxidation in *MMUT* methylmalonic acidemia (MMA) patients: correlations with clinical and biochemical parameters. The lowest 1-^13^C-propionate oxidation rates were mostly observed in *mut* MMA patients, who harbored two loss-of-function (LOF) variants in *MMUT*. Comparisons reached significance for the area under the curve at 120 minutes (adjusted *P* = 0.0154), but with significant overlap with the compound heterozygous carrying LOF/missense or two missense variants. (**b**) 1-^13^C-propionate oxidation rates showed a positive correlation with renal function, with higher propionate oxidation subjects having a near normal estimated glomerular filtration rate (eGFR) (mL/min/1.73 m^2^) calculated with the combined equation using creatinine and cystatin C values (correlation coefficient *r* = 0.436, *R*^*2*^ = 0.1909, *P* = 0.0098). (**c**) Age of onset for stage 3 chronic kidney disease (CKD) (eGFR based on the bedside Schwartz equation ≤60 mL/min/1.73m^2^) was censored in a Kaplan–Meier curve for *mut*^0^ MMA patients stratified by their 1-^13^C-propionate oxidation rate. Fifty percent of patients with the lowest (<10%) propionate oxidation reached stage 3 CKD at age 8.3 years, as opposed to 15 years in patients with oxidation >20% (marked in red and blue, respectively, *P* = 0.0574). (**d**, **e**) Patients with the lowest 1-^13^C-propionate oxidation (<10%, red) had more severe intellectual impairment, based on standardized age-appropriate neurocognitive evaluations (full-scale IQ [FSIQ]) compared with patients with >20% oxidation rates (adjusted *P* = 0.0325, blue). Bivariate correlation coefficient *r* = 0.455, *R*^*2*^ = 0.2078, *P* = 0.0129. (**f–i**) Significant correlations are shown between 1-^13^C-propionate oxidation rates and clinical disease-specific serum biomarkers, including serum methylmalonic acid (*r* = −0.739, *R*^*2*^ = 0.547, *P* < 0.0001, log transformed values were used for skewed variables); acyl/free carnitine ratio (*r* = −0.584, *R*^*2*^ = 0.341, *P* < 0.0004), as well as biomarkers of hepatic or multisystem mitochondrial dysfunction (FGF21, fibroblast growth factor 21: *r* = −0.486, *R*^*2*^ = 0.237, *P* < 0.0064 and GDF15, growth differentiation factor 15: *r* = −0.664, *R*^*2*^ = 0.4418, *P* < 0.0001).

We queried our natural history database to examine whether 1-^13^C-propionate oxidation correlated with canonical biochemical measures and other clinical parameters. We examined associations with molecular genotype, renal function indices, neurocognitive outcomes, growth parameters, and disease-specific biomarkers, parameters that can be tested clinically in most metabolic centers. Results of the nontransplanted *mut* cohort are presented in Fig. 3, while similar analyses in the entire isolated MMA patient cohort, including *cblA* and *cblB* subjects, are provided in Supplemental Fig. 3.

Patients with two loss-of-function (LOF) variants in *MMUT*, predicted to have very little or no protein expression and/or enzymatic function, had the lowest 1-^13^C-propionate isotopic enrichment rates, but there was overlap with compound heterozygotes for LOF and missense or two missense variants (Fig. 3a). Lowest oxidation rates also correlated with impaired renal function (Fig. 3b), one of the major chronic disease complications in *mut* MMA patients. This result could represent an interference of the renal dysfunction with test performance rather than an indication that patients with lower activity have the most severe kidney disease. We therefore stratified patients by 1-^13^C-propionate oxidation rate and examined the age of onset of stage 3 CKD. The lowest 1-^13^C-propionate oxidation (<10%) was associated with a trend for earlier onset of stage 3 CKD in this *mut*^0^ MMA patient cohort, compared with patients oxidizing 10–20% or >20% of the isotopomer in 120 minutes (Kaplan–Meier curve, *P* = 0.0574 comparing <10 with >20% oxidation, Fig. 3c). If cystatin C or combined creatinine and cystatin C eGFR equations were used, the stage 3 CKD would be noted even earlier,^14^ but historic longitudinal data were mostly available for creatinine values for all patients.

Similarly, *mut* MMA subjects with <10% 1-^13^C-propionate oxidation had lower cognitive function compared with subjects with >20% oxidation (*P* = 0.0325), while correlation parameters of the 60-minute 1-^13^C-propionate oxidation with cognitive function were *r* = 0.455, *R*^*2*^ = 0.207, *P* = 0.012 (Fig. 3d–e). Lastly, correlations with several disease-specific metabolites (serum methylmalonic acid, acylcarnitine to free carnitine ratio) and recently described biomarkers of mitochondrial dysfunction (fibroblast growth factor 21 and growth differentiation factor 15) were observed^30,39^ (Fig. 3f–i).

When *cblA* and *cblB* subjects were included in the analysis with the *mut* cohort, correlations could be demonstrated between 1-^13^C-propionate oxidative capacity and growth parameters (height and head circumference *z*-scores) and bone mineral density, in addition to cognitive function, renal disease, and serum biomarkers (Supplemental Fig. 3a–l).

Lastly, in 11 patients, we studied the correlation of 1-^13^C-propionate breath test to in vitro ^14^C-propionate incorporation into protein in skin fibroblasts that were previously obtained for diagnostic purposes (Supplemental Table 1, and marked^c^ in Table 1). Parameters of the 1-^13^C-propionate breath test were correlated to parameters of the fibroblast enzyme activity assays. The percent of control ^14^C-propionate incorporation correlated with the 60-minute 1-^13^C-propionate propionate oxidation (Spearman *r* = 0.682, *R*^*2*^ = 0.327, *P* = 0.025, Supplemental Fig. 3m) noted in patients.

## DISCUSSION

Isolated methylmalonic acidemia encompasses a heterogeneous group of inborn errors of metabolism with a wide spectrum of phenotypic severity, rate of disease progression, and long-term outcomes, especially for patients with the severe MMUT or MMAB enzymatic defects. Genotype–phenotype/enzymatic activity correlations in *mut* MMA are challenging: the majority of patients are compound heterozygotes for rare missense variants with limited functional characterization, and cellular enzymology requires a skin biopsy and specialized radioactivity assays only available in few centers.^4,5,40^ We therefore revisited an approach to characterize in vivo pathway flux in patients that showed promise when first described several decades ago by Walter and Thompson et al.,^17,25,27^ and later extended by Barshop et al.^26^ We optimized the oral bolus 1-^13^C-propionate oxidation method employed by Barshop et al., using a 20-fold reduced dose and parallel REE measurements, in a large cohort of patients with isolated MMA, including a number of liver and/or kidney transplant recipients, and were able to demonstrate that the 1-^13^C-propionate breath test: (1) provides a safe, noninvasive, and reliable measure of the severity of propionate oxidation enzymatic defect; (2) can produce reproducible result despite widely different metabolite pools in patients with changing renal status; (3) shows a robust response to liver transplant and can detect smaller enzymatic activity changes after an isolated kidney transplant or B_12_ therapy when tested pre- and postintervention in individual subjects; and (4) correlates with disease-specific serum biomarkers and clinical outcomes (summarized in graphical abstract).

The 1-^13^C-propionate breath test was well tolerated by patients across all age groups, with the youngest participant in this study being 3.5 years old. Patients with severe autism spectrum disorder, attention deficit hyperactivity disorder, or a severe movement disorder due to previous basal ganglia injury had difficulties with the metabolic cart part of the study, which requires subjects to remain still for over 30 minutes. Despite these challenges, we were able to complete the 1-^13^C-propionate breath test using one or both breath collection methods (EasySampler or Breath ID®). Notably, the Breath ID® device provides a unique ability to obtain real-time measurements of propionate metabolic flux at the bedside, which would greatly facilitate the assessment of pharmacodynamic responses to gene/enzyme replacement clinical trials.

The simple oral bolus isotope method has well-recognized caveats^26^ and previously failed to show correlation with disease severity similar to the gold standard intravenous isotope oxidation studies.^26^ Bioavailability and pharmacokinetics of a small isotopomer dose delivered by the oral route is affected by fasting state, gut mobility, and microbiome differences, but also, importantly, by the dilution of the tracer in a large endogenous metabolite pool and the effects of renal function on its clearance, parameters that can vary greatly between follow-up clinic visits in MMA patients. Moreover, our previous experience with lower than predicted resting energy expenditure assessments in this patient population^31^ raised concerns about the accuracy of estimated VCO_2_ using standard equations (developed in healthy subjects) previously used for the oral bolus stable isotope studies in MMA and PA. We therefore relied upon a metabolic cart prior to each test to measure the VCO_2_ needed to calculate the percent of label oxidized over time, and employed a minimum of 3-hour fast prior to isotope administration to optimize method reproducibility, as suggested by the study of Wagner et al.^28^ Employing a standardized protocol and testing a larger number of patients across a wide spectrum of disease severity allowed us to achieve reproducibility and clinical correlations comparable to the intravenous isotope enrichment methods.^25^ Reproducibility of the 1-^13^C-propionate breath test was superior to the corresponding serum methylmalonic acid values in patients tested repeatedly over a range of time intervals (2 months to 4 years, Fig. 1e–g), suggesting the test is able to perform well in most patients despite vastly different metabolite pools, dietary status, and overall disease state.

Lastly, 1-^13^C-propionate oxidation rate showed correlations with several clinical parameters (cognitive scores, age of renal function decline, growth indices), as well as serum canonical (methylmalonic acid and acyl/free carnitine ratio) and research biomarkers (FGF21 and GDF15). Moreover, we demonstrate a robust response after a liver or liver/kidney transplantation and smaller changes after an isolated kidney transplant (Fig. 2d and g). Patients with very low 1-^13^C-propionate oxidative capacity (<10%), were more likely to carry two nonsense/LOF variants, have lower glomerular filtration rate or earlier onset of stage 3 CKD, a lower FSIQ, as well as shorter stature and decreased bone mineral density. Given the strong correlation of serum methylmalonic acid concentrations with renal function, it is particularly useful to establish additional measures of disease severity and response to liver corrective therapeutic interventions. We have previously shown that massively elevated FGF21 plasma concentrations in MMA patients correlate with oxidative stress markers and branched-chain amino acid deficiencies, but not significantly with renal function, and are completely normalized after liver transplantation.^30^ We propose that the combination of 1-^13^C-propionate oxidative capacity with FGF21 and GDF15 levels will significantly enhance the ability to detect a response to liver-targeted therapies in MMA, as opposed to obtaining serum methylmalonic acid measurements alone.

In conclusion, this work demonstrates that the 1-^13^C-propionate breath test provides a safe, noninvasive bedside assessment of propionate oxidation that can serve along with the patient’s genotype and biomarkers to prognosticate disease severity and evaluate the response to therapies aimed at increasing MMUT or other propionate oxidation pathway enzymes, such as PCCA and PCCB (see part 2 submission). In subsequent studies, we propose to evaluate the use of 1-^13^C-propionate breath test in selecting the severely affected patients that will benefit most from an early referral for a liver transplantation surgery and/or for stratification in clinical trials. Furthermore, this method may provide an objective measure of the pharmacodynamic response and assist in the real-time monitoring of the gains in enzymatic function achieved by small molecule, gene, mRNA, or enzyme replacement strategies.

## Supplementary information

Supplementary information

## Data Availability

Authors are willing to share the data and protocols presented in this work upon request.
