# Correction to: 1-^13^C-propionate breath testing as a surrogate endpoint to assess efficacy of liver-directed therapies in methylmalonic acidemia (MMA)

**DOI:** 10.1038/s41436-021-01188-9

**Published:** 2021-05-10

**Authors:** Irini Manoli, Alexandra R. Pass, Elizabeth A. Harrington, Jennifer L. Sloan, Jack Gagné, Samantha McCoy, Sarah L. Bell, Jacob D. Hattenbach, Brooks P. Leitner, Courtney J. Duckworth, Laura A. Fletcher, Thomas M. Cassimatis, Carolina I. Galarreta, Audrey Thurm, Joseph Snow, Carol Van Ryzin, Susan Ferry, Nicholas Ah Mew, Oleg A. Shchelochkov, Kong Y. Chen, Charles P. Venditti

**Affiliations:** 1 Medical Genomics and Metabolic Genetics Branch, National Human Genome Research Institute, National Institutes of Health, Bethesda, MD USA; 2 Diabetes, Endocrinology, and Obesity Branch, National Institute of Diabetes and Digestive and Kidney Diseases, National Institutes of Health, Bethesda, MD USA; 3 Pediatrics and Developmental Neuroscience Branch, National Institute of Mental Health, National Institutes of Health, Bethesda, MD USA; 4 Office of the Clinical Director, National Institute of Mental Health, National Institutes of Health, Bethesda, MD USA; 5 Children’s National Health System, Washington, DC, USA

Correction to: *Genetics in Medicine* 2021; 10.1038/s41436-021-01143-8; published online 05 April 2021

Due to a processing error, the online graphical abstract was not given.
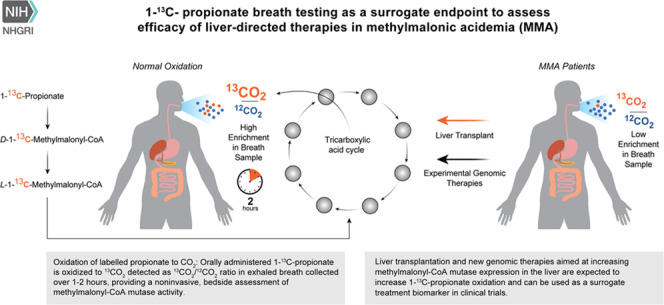


The original article has been corrected.

