# Supplementary material for: 1-13C-propionate breath testing as a surrogate endpoint to assess efficacy of liver-directed therapies in methylmalonic acidemia (MMA)
Source: Genet Med. 2021 Apr 5;23(8):1522–33. doi: 10.1038/s41436-021-01143-8 (PMC8354855; doi:10.1038/s41436-021-01143-8)
Supplement: Supplementary file 1 — Supplementary information [file 41436_2021_1143_MOESM1_ESM.pdf]

Supplemental Figure 1

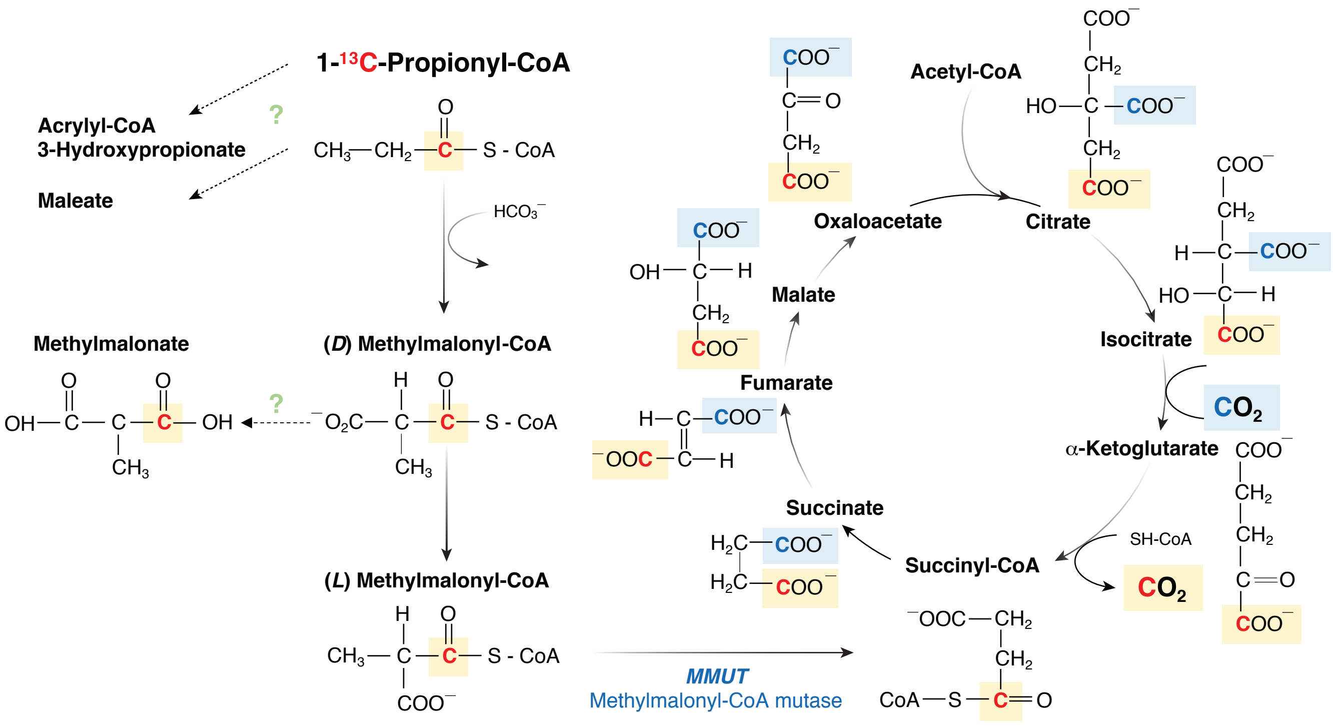

Supplemental Figure 2

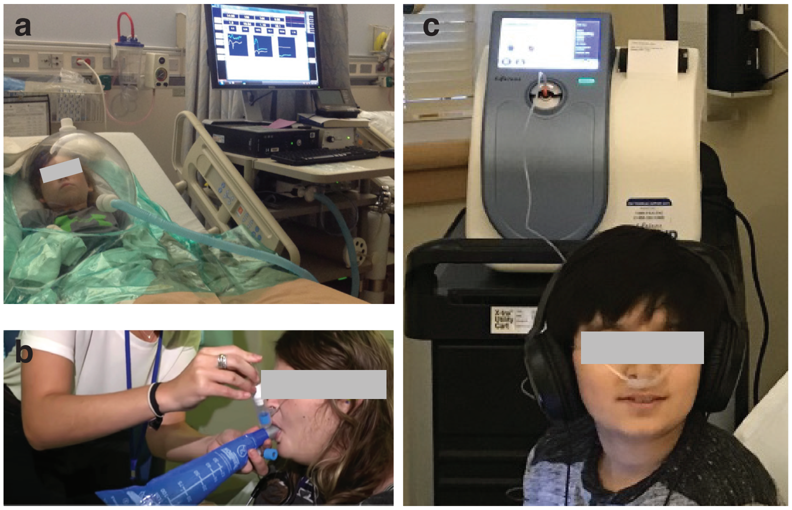

Supplemental Figure 3

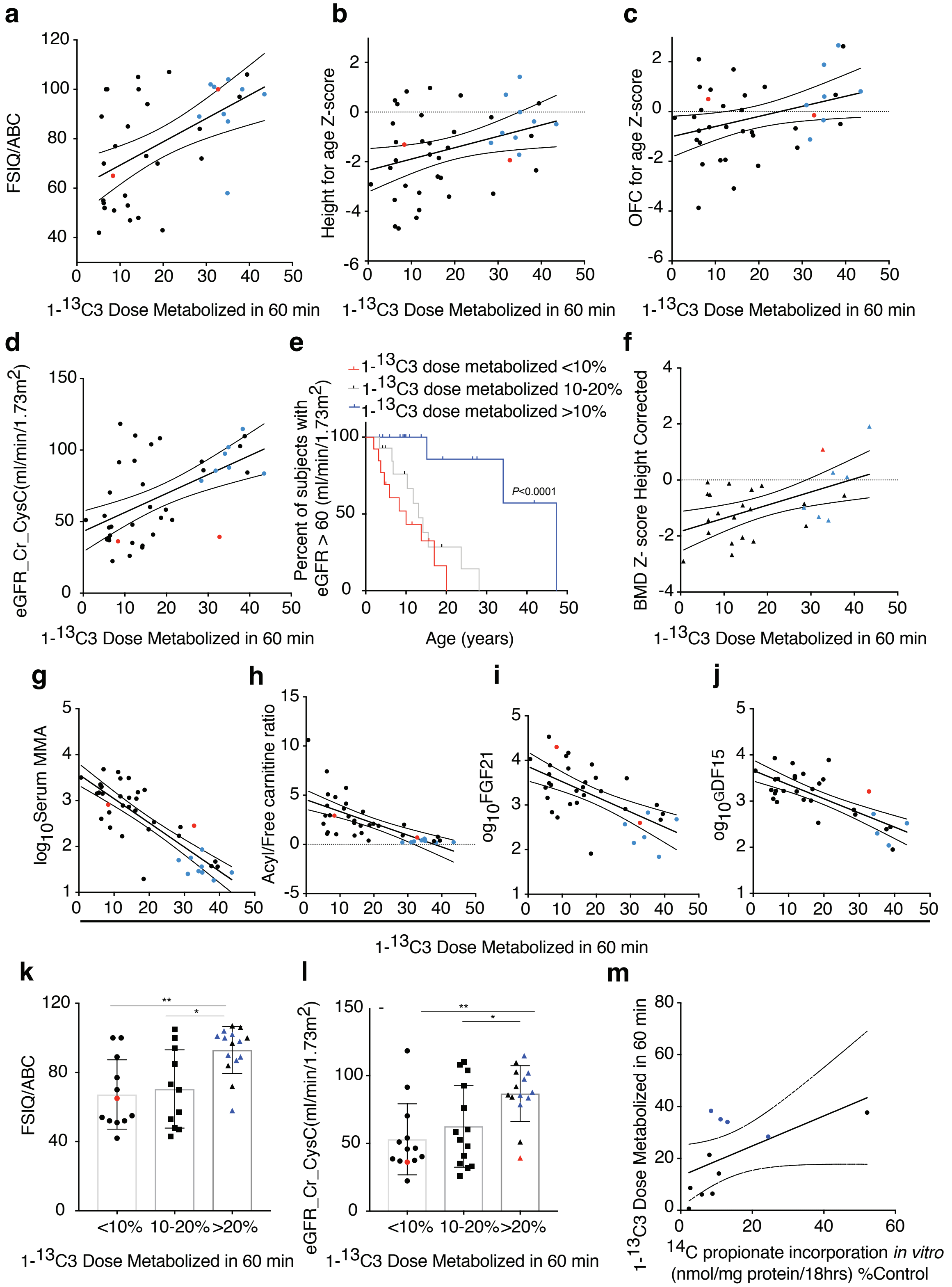

Supplemental table 1: Comparison of 1-^{13}C3 breath test with diagnostic ^{14}C3 fibroblast enzyme assays

#	Gene	Variant 1	Variant 2	% 1-^{13}C3 dose metabolized (60 and 120min)		AUC 1-^{13}C3 dose metabolized (60 and 120min)		Tmax Δ^{13}C (min)	Cell line WG #	Fibroblast ^{14}C3 Uptake (nmol/mg prot/18h)	Fibroblast ^{14}C3 Uptake %control	Fibroblast +OHCbl % baseline
12	*MMUT*	c.1741C>T, p.Arg581Ter	c.753+2T>A, p.splice	.63	7.20	4.73	235.3	90	2883	.26	2.28	26.92
17	*MMUT*	c.682C>T, p.Arg228Ter	c.607G>A, p.Gly203Arg	8.62	20.24	235.9	1177.0	41	3231	.22	2.65	15.53
22	*MMUT*	c.29dupT, p.Leu11Thrfs*38	c.1658delT, p.Val553Glyfs*17	6.12	15.09	125.4	769.0	60	3426	.50	5.88	.00
15	*MMUT*	c.91C>T, p.Arg31Ter	c.2053_2055dupCTC p.Leu685dup	21.38	41.37	497.2	2430.0	45	2670	.96	8.00	35.41
3	*MMUT*	c.682C>T, p.Arg228Ter	c.322C>T, p.Arg108Cys	6.43	13.92	230.3	920.0	25	3529	1.40	8.86	7.14
13	*MMUT*	c.1942G>C, p.Gly642Arg	c.1942G>C, p.Gly642Arg	14.19	31.25	297.7	1698.0	45	2839	1.60	10.66	12.50
31	*MMUT*	c.1181T>A, p.Leu394Ter	c.1942G>C, p.Gly642Arg	37.70	48.35	1165.0	4330.0	20	3504	7.50	52.08	-1.33
36	*MMAA*	c.433C>T, p.Arg145Ter	c.1075C>T, p.Arg359Ter	38.33	54.39	1201.0	4037.0	15	3106	1.10	8.46	354.54
34	*MMAA*	c.387C>A, p.Tyr129Ter	c.592_595delACTG p. Thr198Serfs*6	35.10	51.79	929.2	3611.0	30	4089	1.20	11.11	258.33
40	*MMAA*	c.433C>T, p.Arg145Ter	c.433C>T, p.Arg145Ter	34.04	52.46	1005.0	3642.0	25	3490	1.80	13.04	544.40
39	*MMAA*	c.440G>A, p.Glu147Gly	c.450dupG, p.Pro151Alafs*19	28.38	40.65	741.5	2876.0	30	1518	4.45	24.45	151.68

Supplemental Figure 1. An overview of propionyl-CoA oxidation pathway. The biochemical pathway of 1-^{13}C- labeled propionyl-CoA oxidation is depicted, following the fate of the heavy labeled carbon administered for the 1-^{13}C-propionate breath test through the methylmalonyl-CoA mutase reaction and subsequently the Krebs or tricarboxylic acid (TCA) cycle, until its release as $^{13}CO_2$. The heavy carbon is colored in red. $^{13}CO_2$ can be released at the α-ketoglutarate dehydrogenase or at the succinyl-CoA synthetase steps depending on the position of the heavy carbon after the carbon skeleton rearrangement during the racemization of L-methylmalonyl-CoA to syccinyl-CoA before entering the TCA cycle. The fate of administered 1-^{13}C-propionate is likely modified by several factors, such as CoA trapping[1], acrylyl-CoA and maleate synthesis or other alternative propionate decarboxylation pathways, as well as isotope exchange between the 1-C and 3-C carbons of methylmalonyl-CoA and free methylmalonic acid[2,3]. Moreover, variable secondary impairments in the activity of the TCA cycle could affect the 1-^{13}C-propionate oxidation results and test reproducibility. Performing a parallel study of 1-^{13}C -acetate oxidation into bicarbonate and exhaled $^{13}CO_2$ would allow correcting the propionate oxidation calculations by the acetate recovery factor[4]. It was difficult to implement two consecutive fasting studies in one patient visit in this unstable patient population and this was not further pursued. Despite these theoretical considerations, the performance of the 1-^{13}C-propionate breath test in the current study, suggests that variations introduced by these additional pathways to the propionate flux in the MMA patient cohort did not significantly affect method reproducibility.

Supplemental Figure 2. Metabolic cart and breath collection methodology.

(**a**) An open-circuit indirect calorimetry method (ventilated hood) is used prior to the 1-^{13}C-propionate breath test to measure O_2 consumption (VO_2), CO_2 production (VCO_2), and basal or resting energy expenditure (REE). Patient is lying supine in bed for 30 min to achieve a steady

state baseline followed by the metabolic cart measurements for about 20-30 min. The flow rate of the open-circuit system was set between 20-30 L/min to achieve 0.9-1.2% end-tidal CO_2 concentration, which is the optimal sensitivity range for the near-infrared CO_2 analyzer and with minimal impact on subject's normal breathing patterns. Before each test, the calorimeter was calibrated with the use of a reference gas mixture obtained from the manufacturer. A computer terminal is used at the bedside to ensure quality data collection throughout the test. (**b**) Breath collection for the measurements of $^{13}CO_2/^{12}CO_2$ enrichment in exhaled air over time is achieved by direct collection of breath samples in vacutainer tubes using disposable collection kits where the patient blows in a balloon, with adjustment of the balloon volume by weight per manufacturer's instructions (EasySampler™ Breath Test Kit, Quintron). (**c**) The BreathID® Exalenz device senses exhaled breath continuously through a nasal cannula worn by the patient and measures $^{13}CO_2$ and $^{12}CO_2$ concentrations every 4 minutes to establish the $^{13}CO_2/^{12}CO_2$ ratio prior and after test substrate (1-^{13}C-propionate) administration. Wearing the nasal canula, patients establish a baseline level of $^{13}CO_2$. Once the baseline was established, the patients ingested the isotope and remained connected to the machine for up to 2 hours. Molecular Correlation Spectrometry (MCS™) utilized by the BreathID® device MCS technology measures the light absorbance of the sample by infrared spectrometry; the software provides real time results as the difference between the ratio of $^{13}CO_2$ /$^{12}CO_2$ in the test specimen and the corresponding baseline sample (Delta Over Baseline). Results are depicted in real time and printed at the patient bedside.

Supplemental Figure 3. 1-^{13}C-propionate oxidation in isolated MMA: correlations with clinical and biochemical parameters (**a-d**) 1-^{13}C-propionate oxidation at 60 min correlates with several additional quantitative measures of clinical outcomes, when subjects with *cblA* and *cblB* isolated MMA subtypes are added to the analysis. Subjects with cblA, the B_{12} responsive

subtype of MMA, are depicted with **blue** symbols and cblB with **red** symbols in all the graphs. Cognitive function, expressed as Full Scale IQ and Adaptive Behavior Composite (correlation coefficient: r=0.533, R^2=0.284, P=0.0006); height for age Z-score (r=0.352, R^2=0.124, P=0.0205); occipitofrontal head circumference Z-score (r=0.351, R^2=0.123, P=0.0284); eGFR calculated using the CKID combined creatinine and cystatin-C equation[5] (r=0.555, R^2=0.309, P=0.0002). (**e**) Kaplan-Meier curve showing the rate of decline to Stage 3 chronic kidney disease (estimated GFR based on the bedside Schwartz equation ≤60mL/min/1.73m^2) is shown in patients stratified by their 1-^{13}C-propionate oxidation rate. 50% of patients with the lowest (<10%) propionate oxidation reached Stage 3 CKD at age 8.3 years, as opposed to age 11.8 and 34 years for patients with oxidation rates 10-20 and >20% (P<0.0001). (**f**) Bone mineral density Z-scores corrected for the short stature of MMA patients also showed a positive correlation with propionate oxidation rates at 60 min (r=0.527, R^2=0.278, P=0.0047). (**g-j**) Correlations of 1-^{13}C-propionate oxidation at 60 min with serum biomarkers is shown for methylmalonic acid (variables with skewed distributions were log transformed, correlation coefficient r= -0.6439, R^2=0.414, P<0.0001 for serum MMA, or r= -0.847, R^2=0.717, P<0.0001 for logMMA); acyl/free carnitine ratio (r= -0.680, R^2=0.462, P=0.0006); logFGF21 (r= -0.637, R^2=0.405, P<0.0001) and logGDF15 (r= -0.748, R^2=0.560, P<0.0001). (**k-l**) Cognitive function and estimated GFR values are shown for ranges of 1-13C propionate oxidation (**P < 0.01, *P < 0.05). (**m**) A small subset of patients had a skin biopsy for *in vitro* fibroblast assays using radioactive ^{14}C-propionate uptake/incorporation for diagnostic purposes (**Supplemental Table 1**). Correlation with the 1-^{13}C breath test results is depicted, Spearman correlation r=0.682, R^2=0.327, P=0.025. Patients with *cblA* subtype are denoted by blue symbols.

References in Supplemental Figures:

1. Yang, H. *et al.* Inborn errors of mitochondrial acyl-coenzyme a metabolism: acyl-CoA biology meets the clinic. *Mol Genet Metab* **128**, 30-44 (2019).
2. Wilson, K.A. *et al.* Inter-relations between 3-hydroxypropionate and propionate metabolism in rat liver: relevance to disorders of propionyl-CoA metabolism. *Am J Physiol Endocrinol Metab* **313**, E413-E428 (2017).
3. Montgomery, J.A., Mamer, O.A. & Scriver, C.R. Metabolism of methylmalonic acid in rats. Is methylmalonyl-coenzyme a racemase deficiency symptomatic in man? *J Clin Invest* **72**, 1937-47 (1983).
4. Schrauwen, P., Blaak, E.E., Van Aggel-Leijssen, D.P., Borghouts, L.B. & Wagenmakers, A.J. Determinants of the acetate recovery factor: implications for estimation of [13C]substrate oxidation. *Clin Sci (Lond)* **98**, 587-92 (2000).
5. Schwartz, G.J. *et al.* Improved equations estimating GFR in children with chronic kidney disease using an immunonephelometric determination of cystatin C. *Kidney Int* **82**, 445-53 (2012).
